# Evaluation of association of *DRD2 Taq*IA and *-141C InsDel* polymorphisms with food intake and anthropometric data in children at the first stages of development

**DOI:** 10.1590/1678-4685-GMB-2017-0202

**Published:** 2018-07-23

**Authors:** Vanessa Feistauer, Márcia R. Vitolo, Paula D.B. Campagnolo, Vanessa S. Mattevi, Silvana Almeida

**Affiliations:** 1 Laboratório de Biologia Molecular, Programa de Pós-Graduação em Biociências, Universidade Federal de Ciências da Saúde de Porto Alegre, Porto Alegre, RS, Brazil; 2 Departamento de Saúde Coletiva, Universidade Federal de Ciências da Saúde de Porto Alegre, Porto Alegre, RS, Brazil; 3 Curso de Nutrição, Universidade do Vale do Rio dos Sinos, São Leopoldo, RS, Brazil; 4 Departamento de Ciências Básicas da Saúde, Universidade Federal de Ciências da Saúde de Porto Alegre, Porto Alegre, RS, Brazil

**Keywords:** Child obesity, DRD2 polymorphisms, food intake

## Abstract

The reward sensation after food intake may be different between individuals and variants in genes related to the dopaminergic system may indicate a different response in people exposed to the same environmental factors. This study investigated the association of *Taq*IA (rs1800497) and *-141C InsDel* (rs1799732) variants in *DRD2/ANKK1* gene with food intake and adiposity parameters in a cohort of children. The sample consisted of 270 children followed until 7 to 8 years old. DNA was extracted from blood and polymorphisms were detected by PCR-RFLP analysis. Food intake and nutritional status were compared among individuals with different SNP genotypes. Children carrying the *A1* allele (*Taq*IA) had higher energy of lipid dense foods (LDF) when compared with *A2/A2* homozygous children at 7 to 8 years old (GLM *p*=0.004; Mann Whitney *p*=0.005). No association was detected with *-141C Ins/Del* polymorphism. To our knowledge, this is the first association study of the *DRD2 Taq*IA and *-141C Ins/Del* polymorphism with food intake and anthropometric parameters in children. *DRD2 Taq*IA polymorphism has been associated with a reduction in D_2_ dopamine receptor availability. Therefore, the differences observed in LDF intake in our sample may occur as an effort to compensate the hypodopaminergic functioning.

## Introduction

The prevalence of childhood overweight and obesity had a dramatic increase between 1990 and 2010, rising from 4.2% to 6.7%, and it is estimated that in 2020 the rate will be 9.1%, or approximately 60 million children (de Onis *et al.*, 2010). The obesity prevalence in developed countries is twice higher than in developing countries. However, most of the affected children (35 million) live in developing countries (de Onis *et al.*, 2010). Moreover, the relative increase rate of obesity in recent decades was higher in developing countries (+65%) than in developed countries (+48%) (de Onis *et al.*, 2010; Oggioni *et al.*, 2014). Obese children are more likely to become obese adults, and have higher risk of developing coronary heart diseases and other related diseases, which diminish life expectancy (Must, 1996; Rossner, 1998; Berenson, 2012). Insulin resistance, metabolic syndrome, and type 2 diabetes are also consequences of childhood obesity (Gupta *et al.*, 2012).

Some factors contribute to overweight and obesity, such as low physical activity, high intake of high fat and sugar foods, change from the rural lifestyle to the urban, sociocultural factors, age, gender, and genetic factors (Popkin, 2006; Gupta *et al.*, 2012; Oggioni *et al.*, 2014). The sensation of reward after food intake, especially of palatable foods, may be different among individuals and might cause different amounts of food ingestion (Berridge *et al.*, 2010). The dopaminergic system regulates food intake through a reward system, and although its function in eating disorders is poorly understood, it is known that the use of dopamine D_2_ receptors agonists decreases food intake in rats (Terry *et al.*, 1995). A study that analyzed images via Positron Emission Tomography (PET) scans shows that obese individuals have low concentration of striatal D2 dopamine receptors as a mechanism of downregulation due to high levels of dopamine, indicating that the reduction of these receptors could be associated with an addictive behavior also observed in drug users (Wang *et al.*, 2001). *DRD2/ANKK1* gene polymorphisms alter the density of dopamine receptors, and thus may explain the different food intake levels in individuals exposed to the same environmental factors (Stelzel *et al.*, 2010).

Several studies have associated the *Taq*IA (rs1800497) polymorphism with obesity, body mass index (BMI), and food intake (Barnard *et al.*, 2009; Winkler *et al.*, 2012; Cameron *et al.*, 2013; Carpenter *et al.*, 2013). However, to our knowledge, there is no study linking the *-141C Ins/Del* (rs1799732) polymorphism to obesity, although it was associated with other pathologies such as alcoholism and schizophrenia (Jonsson *et al.*, 1999a; Johann *et al.*, 2005; Lafuente *et al.*, 2008a,b). Therefore, the objective of the present study was to analyze the association of *Taq*IA and *-141C Ins/Del* polymorphisms with adiposity parameters and food intake of children.

## Materials and Methods

### Subjects

The sample consisted of 270 children followed until 7 to 8 years old on average. The nutritional and anthropometrics data were collected at 12 to 16 months, 3 to 4 and 7 to 8 years. The children included in the present study participated in a randomized controlled trial of dietary counseling on breast feeding and diet during the first year of life. The trial consisted of 500 children, randomized in a control or intervention group, of which mothers received a dietary advice about breastfeeding and complementary feeding during home visits in children’s first year of life. This dietary advice was based on the “Ten steps to Healthy Feeding”, a Brazilian national health policy for primary care, supported by World Health Organization (2006). More information of the first phase of the study can be found elsewhere (Vitolo *et al.*, 2010), but in Table 1 we described the main characteristics of the sample. A substantial reduction of the sample occurred throughout the study and the main reason for the losses was the inability to locate the participants’ homes, usually due to the family moving to another city. Other reasons for losses were refusal to continue and children or maternal death. This intervention was not the primary objective of the present research and the participation in the intervention or control group was used as a confounding factor in statistical analyses.

**Table 1:** Main characteristics of the sample.

Characteristics	
Ethnicity (whites) ^a^	210 (77.8)
Sex (boys)^a^	149 (55.2)
**12 to 16 months**	
Child’s BMI (Z-score) ^b^	0.59 ± 1.07
Average daily energy intake (kcal)^b^	948.51 ± 398.56
**3 to 4 years**	
Child’s BMI (Z-score) ^b^	0.27 ± 1.16
Average daily energy intake (kcal) ^b^	1520.21 ± 391.36
Sugar dense food intake (SDF; kcal) ^b^	105.94 ± 86.15
Lipid dense food intake (LDF; kcal) ^b^	177.18 ± 190.81
Average daily energy intake per kilogram (kcal/kg)^b^	91.23 ± 27.27
**7 to 8 years**	
Child’s BMI (Z-score)^b^	0.41 ± 1.38
Average daily energy intake (kcal)^b^	1564.54 ± 381.34
Sugar dense food intake (SDF; kcal)^b^	230.02 ± 194.97
Lipid dense food intake (LDF; kcal)^b^	85.10 ± 80.33
Average daily energy intake per kilogram (kcal/kg)^b^	59.75 ± 18.10

a Data are presented as % (n).

b Data are presented as mean ± standard deviation.

Ethnicity was defined by the interviewer by skin color (i.e., whites and non-whites). More details of the traits studied are described elsewhere (Galvão, 2012; Louzada *et al.*, 2012; Fontana *et al.*, 2015; Miranda *et al.*, 2015). This study was conducted according to the guidelines of the Declaration of Helsinki. The study protocol was approved by the Ethics Committee of the Universidade Federal de Ciências da Saúde de Porto Alegre (n. 286/06), and all participants provided written informed consent before commencing the study.

### Nutritional status assessment

At 12 to 26 months, children were weighted using a portable digital scale (Techline, São Paulo, Brazil) and length was measured by an infant stadiometer (Serwital Inc, Porto Alegre, Brazil). At 3 to 4 and 7 to 8 years, children were weighted using a digital scale (Techline), and height was measured using a stadiometer (SECA, Hamburg, Germany). BMI was calculated [weight (kg)/height^2^(m^2^)], and the values were transformed into Z-scores.

### Dietary data assessment

One 24-hour dietary recall was collected for each child at 12 to 16 months, and two 24-hour dietary recalls, on two nonconsecutive days, were collected for each child at the ages of 3 to 4 and 7 to 8 years. The 24-hour dietary recall was carried out by a trained undergraduate nutrition student, and the child’s food intake was recorded on the day before the last home visit. A food portion measurement device and the common household measures (e.g. teaspoons, tablespoons, cups) were used to quantify portion sizes.

Dietary information was entered into the Nutrition Support Program software from the Escola Paulista de Medicina, Federal University of São Paulo, based on the United States Department of Agriculture chemical composition tables. The energy intake was calculated using only one dietary recall or the average of two dietary recalls. The items listed in the response were classified as sugar-dense foods (SDF) if the percentage of simple carbohydrates was higher than 50% (e.g., soda, Jell-O, candies, and artificially flavored juice) and as lipid-dense foods (LDF) if there was more than 30% fat (e.g. fried pastries, cookies with fillings, cold cuts and sausages, fried foods, and chocolate).

### DNA analyses

Blood samples for DNA extraction were collected in EDTA tubes (6 mL). Genomic DNA was extracted from peripheral blood leukocytes by the Lahiri and Nurnberger procedure (Lahiri and Nurnberger, 1991). *DRD2/ANKK1 Taq*IA (rs1800497) and *-141C InsDel* (rs1799732) polymorphisms were detected by PCR-RFLP analysis using primer sequences and conditions described by Grandy *et al.* (1993) and Ohara *et al.* (1998), respectively. Primers sequences (IDT Coralville, IA, USA) were as follows: *Taq*IA, forward primer 5’- CACCTTCCTGAGTGTCAT CAA -3’ and reverse primer 5’-AGACAACTTGGCCAG CCGTG-3’; *-141C InsDel*, forward primer 5’-ACTGGC GAGCAGACGGTGAGG and reverse primer 5’-TGCGCGCGTGAGGCTGCCGGT. PCR products were digested separately with either *Taq*I (*Taq*IA polymorphism) or *Mva*I (*-141C InsDel* polymorphism*)* enzyme (Fermentas, Glen Burnie, MD, USA), according to the manufacturer’s instructions. Genotypes were determined after electrophoresis in 2 or 3% agarose gels that had been stained with ethidium bromide. For the *DRD2/ANKK1 Taq*IA polymorphism, the *C* allele contains a *Taq*I restriction site and is designated as the *A2* allele, while the *T* allele is designated as the *A1* allele. For the *-141C InsDel* polymorphism, the -141C*Ins allele contains a restriction site for *Mva*I while the -141C*Del allele does not.

### Statistical analyses

Allele frequencies were estimated by gene counting. A chi-square test for goodness-of-fit was used to determine whether the observed genotype frequency distributions agreed with those expected under Hardy-Weinberg equilibrium. Linkage disequilibrium was estimated using the Haploview software Version 4.2 (Broad Institute, Barrett *et al.*, 2005).

Pearson’s chi-squared or Fisher’s Exact Test was used to compare genotype or allele frequencies between white and non-white children. Since the first publication of an association study with the *Taq*IA polymorphism (Blum *et al.*, 1990), and because of the rare occurrence of the *A1* allele, genotypes are normally grouped as *A1* allele presence or *A1* allele carriers (*A1/A1* and *A1/A2*, n=102), *versus A2/A2* homozygotes (n=116). Similarly, due to low frequencies of the *Del* allele, genotypes of the *-141C InsDel* polymorphism were grouped by *Del* allele presence or *Del* allele carriers (*Del/Del* and *Ins/Del*, n=61), *versus Ins/Ins* homozygotes (n=157). All data are presented as mean and standard deviation. Statistical analysis of SDF and LDF variables were performed on natural logarithm transformed data to normalize their distribution. This allowed including these variables in multivariate analysis; non-transformed values are shown in Table 2. Means of food intake (average daily energy intake, SDF, LDF and average daily energy intake per kilogram) and adiposity (BMI Z-score) parameters were compared among genotype groups by a multivariate general linear model (GLM). The multivariate GLM was performed including all dependent continuous variables in one model, using the categorical variables (1) the control or intervention variable of the randomized trial, (2) sex, and (3) ethnicity as covariates, and genotypes of *-141C InsDel* (rs1799732) and *Taq*IA (rs1800497) polymorphisms as fixed factors (see Table 2). This first step of the analysis verified whether the group of dependent continuous variables was significantly affected by the group of independent categorical variables. Only LDF intake at 7 to 8 years old was associated with *Taq*IA polymorphism, and the covariates did not influence this dependent variable. Therefore, to test the association of *Taq*IA polymorphism alone with LDF intake at 7 to 8 years, we performed a Mann-Whitney test. A *p*-value of < 0.05 was considered significant. All tests and transformations were performed using the Statistical Package for Social Sciences, Version 20.0 (SPSS^®^, Chicago, IL, USA).

**Table 2:** Food intake and anthropometrics parameters according to polymorphisms in *DRD2/ANKK1* gene (*-141C Ins/Del* and *Taq*IA) in children at 1, 3 to 4, and 7 to 8 years old.

	*-141C Ins/Del*			*Taq*IA		
	Genotypes			Genotypes		
	*Ins/Ins* (n=157)	*Del* carriers (n=61)	*p*	*A1* carriers(n=102)	*A2A2* (n=116)	*p*
**12 to 16 months**						
Average daily energy intake (kcal)	930.95 ± 400.09	957.82 ± 390.89	0.601	917.78 ± 395.82	956.66 ± 398.51	0.845
BMI Z-score	0.60 ± 1.13	0.48 ± 1.04	0.609	0.52 ± 1.05	0.62 ± 1.15	0.436
**3 to 4 years**						
Average daily energy intake (kcal)	1506.24 ± 396.82	1579.95 ± 419.60	0.175	1476.62 ± 337.72	1571.04 ± 450.73	0.453
SDF (kcal) ^a^	112.00 ± 97.91	110.77 ± 77.42	0.969	110.0 ± 86.95	113.11 ± 97.40	0.623
LDF (kcal) ^a^	191.55 ± 197.10	162.20 ± 189.60	0.288	168.83 ± 179.06	196.09 ± 208.03	0.884
BMI Z-score	0.27 ± 0.96	0.28 ± 1.62	0.839	0.29 ± 1.17	0.25 ± 1.19	0.774
Average daily energy intake per kilogram (kcal/kg)	91.92 ± 27.74	94.84 ± 29.75	0.448	89.43 ± 24.96	95.65 ± 30.71	0.327
**7 to 8 years**						
Average daily energy intake (kcal)	1560.73 ± 355.60	1581.21 ± 414.36	0.756	1581.13 ± 354.41	1553.56 ± 388.07	0.481
SDF (kcal) ^a^	90.52 ± 75.95	68.03 ± 58.51	0.164	76.97 ± 62.02	90.60 ± 79.62	0.847
LDF (kcal) ^a^	238.38 ± 203.75	223.48 ± 181.94	0.782	282.85 ± 207.96	191.77 ± 178.34	0.004^b^
BMI Z-score	0.45 ± 1.26	0.25 ± 1.65	0.514	0.50 ± 1.36	0.30 ± 1.40	0.258
Average daily energy intake per kilogram (kcal/kg)	59.99 ± 17.00	60.97 ± 20.91	0.861	59.83 ± 17.45	60.65 ± 18.78	0.739

Data are presented as mean ± standard deviation.

BMI – body mass index. SDF – sugar dense foods. LDF – lipid dense foods. General Linear Model (GLM) multivariates with the following co-variates: group (intervention or control), sex, ethnicity.

a Statistical analysis from logarithm form, non-transformed values are shown;

b Mann-Whitney test *p*=0.005

## Results

This longitudinal survey sample was composed of 270 children, 149 (55.2%) boys and 121 (44.8%) girls, followed up from 12 to 16 months until 7 to 8 years old (Table 1). Minor allele frequencies (MAF) of the *DRD2/ANKK1* gene variants observed in the sample were 0.14 of *Del* allele of the *-141C InsDel* (rs1799732) polymorphism and 0.28 of *A1* allele of the *Taq*IA (rs1800497) polymorphism, which were intermediary to those described in the 1000 Genomes Project database for European (MAF 0.08 (*Del*) and 0.19 (*A1*)) and African (MAF 0.57 (*Del*) and 0.38 (*A1*)) populations. All genotype frequencies in this sample were in agreement with those expected under the Hardy-Weinberg equilibrium. The two gene variants were not in linkage disequilibrium (D’=0.3, r=0).

In Table 2, anthropometric and food intake variables are shown according to the analyzed polymorphisms. As some children could not be found at the third home visit at 7 to 8 years, and some samples could not be analyzed in the laboratory, the total number of children included in the multivariate analysis is different from the initial sample size. Children carrying the *A1* allele (*Taq*IA rs1800497) had higher energy of LDF when compared with *A2/A2* homozygous children at 7 to 8 years old (multivariate GLM *p*=0.004; Mann-Whitney test *p*=0.005). No association was detected among *DRD2 -141C Ins/Del* polymorphism with food intake and anthropometric parameters.

## Discussion

The dopaminergic pathway has been associated with midbrain reward circuit activation (Roth *et al.*, 2013), and individual differences in D_2_ receptor expression are hypothesized to contribute to differences in motivated behaviors, such as the motivation to eat (Gluskin and Mickey, 2016). Therefore, polymorphisms of the *ANKK1/DRD2* gene are frequently associated with altered perception of food reward and weight gain (Ariza *et al.*, 2012; Muller *et al.*, 2012; Roth *et al.*, 2013). *Taq*IA is the most commonly tested polymorphism, and is characterized by a single nucleotide change [C(*A2*)/T(*A1*)] located downstream of the termination codon of *DRD2* gene at the *ankyrin repeat and kinase domain containing 1* (*ANKK1)* gene (Dubertret *et al.*, 2004; Neville *et al.*, 2004; Li *et al.*, 2015; Ponce *et al.*, 2016). This SNP produces a Glu713-to-Lys (E713K) substitution in the ANKK1 amino acid sequence, at the eleventh ankyrin, which may alter the affinity of the ANKK1 protein and its substrate (Neville *et al.*, 2004). It is not clear by which molecular mechanisms the ANKK1 protein could be associated with the dopaminergic system and how *ANKK1* polymorphic alleles would impact addiction vulnerability. However, *ANKK1* and *DRD2* genes belong to the same gene cluster, the NTAD cluster, an ancient cluster of which genes are apparently co-regulated and may have emerged when the central nervous system became more complex (Mota *et al.*, 2012). Since genes of related function are sometimes found in the same cluster, it is possible that ANKK1 is somehow involved in the dopaminergic reward processes via a signal transduction pathway (or other cellular response) (Neville *et al.*, 2004). A few *in vitro* studies with *ANKK1* gene mRNAs and proteins were able to show a potential connection between this gene and the dopaminergic system (Hoenicka *et al.*, 2007; Garrido *et al.*, 2011).

In our sample, the *A1* allele (*Taq*IA rs1800497) was found associated with higher intake of LDF when compared with children *A2/A2* homozygous at 7 to 8 years. This allele has been associated with a reduction in D_2_ receptor availability (Pohjalainen *et al.*, 1998; Ritchie and Noble, 2003; Eisenstein *et al.*, 2016). Stice *et al.* (2008) found that the dorsal striatum is less responsive to food reward in obese relative to lean individuals, probably because obese individuals have reduced D_2_ receptor density that compromises dopamine signaling. This hypodopaminergic functioning or reward deficiency syndrome may induce obese patients to overeat in an effort to compensate for this reward deficit; several studies are consistent with this theory (van Strien *et al.*, 2010; Duran-Gonzalez *et al.*, 2011; Winkler *et al.*, 2012; Cameron *et al.*, 2013). van Strien *et al.* (2010) associated the *A1* allele with an increase in emotional eating in Dutch adolescents. The *A1* allele was also most frequent in young obese Mexican-American subjects than in non-obese, as well as subjects with central-obesity versus subjects with no central-obesity (Duran-Gonzalez *et al.*, 2011). Winkler *et al.* (2012) observed in an intervention study that carriers of the *A1* allele had a higher BMI at all time-points (baseline, after weight loss, and after weight maintenance), and showed less overall weight loss. Similarly, Cameron *et al.* (2013) observed that post-menopausal women carriers of the *A1* allele lost significantly less body weight and fat mass than women with the *A2/A2* genotype after undergoing an intervention-induced weight loss and increased carbohydrate intake. Some studies were not able to find any association of the *DRD2 Taq*IA polymorphism with adiposity parameters (Hardman *et al.*, 2014).

In the present study, no association was detected between *DRD2 -141C Ins/Del* polymorphism with food intake and anthropometric parameters, despite previous findings relating *Del* carriers of the *DRD2 -141C Ins/Del* polymorphism with higher D_2_ receptor density (Jonsson *et al.*, 1999b). The *DRD2 -141C Ins/Del* polymorphism corresponds to a deletion of one cytosine from a run of two cytosines at position -141 of the *DRD2* gene (Arinami *et al.*, 1997). This polymorphism has been associated with risk of schizophrenia in different populations (Arinami *et al.*, 1997; Ohara *et al.*, 1998; Jonsson *et al.*, 1999a; Himei *et al.*, 2002; Wu *et al.*, 2005; Lafuente *et al.*, 2008a,b; Cordeiro *et al.*, 2009; Saiz *et al.*, 2010; Xiao *et al.*, 2013; Wang *et al.*, 2016; Zhao *et al.*, 2016), as well as with weight gain (Lencz *et al.*, 2010) and other responses due to schizophrenia drug treatment (Lencz *et al.*, 2006; Zhang *et al.*, 2010). Associations have been described with propensity to alcohol dependence in different populations (Ishiguro *et al.*, 1998; Konishi *et al.*, 2004a,b; Johann *et al.*, 2005; Du and Wan, 2009; Prasad *et al.*, 2010; Lee *et al.*, 2013), suicide attempts (Suda *et al.*, 2009), psychiatric disorders (Kishida *et al.*, 2004; Ujike *et al.*, 2009; Lencer *et al.*, 2014), different responses to medication and higher quit rates in smokers (Lerman *et al.*, 2006). To the best of our knowledge, there is no other study that associated the *DRD2 -141C Ins/Del* polymorphism with anthropometric parameters or food intake.

The lack of associations in the two other phases of development (12 to 16 months and 3 to 4 years) may have occurred because children at these ages have restricted access to food, and depend on adults for meals, despite their own preferences. Notwithstanding, at 7 to 8 years, children have many opportunities to eat without parental supervision (Briefel *et al.*, 2009), and the differences observed in LDF intake in our sample may have occurrred as an effort to compensate hypodopaminergic functioning.

Palatability is the induced sensitive response of foods that are usually rich in lipids and/or sugar (Cansell and Luquet, 2016). The sense of taste during food ingestion is the most important aspect in the decision to consume or avoid foods (Besnard, 2016). Contrary to sugar, oral fat perception was considered dependent only on its textural and olfactory cues, but recent identification of lipid-receptors in taste buds of both rodents and humans strongly suggests that lipids might also be perceived by the gustatory pathway (Besnard, 2016). Stimulation of taste buds triggers a signaling cascade leading to subsequent neurotransmitter releases in different brain areas responsible for taste perception (e.g., anterior insula, frontal operculum, orbitofrontal cortex, and the mesolimbic system) (Besnard, 2016). The exchange between these areas results in information of the hedonic experience related to the food’s taste (Berridge, 1996). Therefore, not only sugar, but also lipids generate a hedonic experience, producing a positive reinforcement that stimulates dopamine secretion in the brain (Salamone, 1994, Volkow *et al.*, 2002), which is a stimulus associated with “wanting” (Berridge *et al.*, 2010). “Wanting” is an incentive salience or motivation for reward triggered by reward-related cues, such as LDF (Berridge *et al.*, 2010). The attribution of incentive salience makes a cue and its reward more attractive, or more “wanted”, without being necessarily more “liked” (Berridge *et al.*, 2010). Consistent with our findings, other studies of our group detected associations of palatable food intake with another polymorphism related to the dopaminergic system in children of the same cohort at 12 to 16 months and 3 to 4 years old (Galvão *et al.*, 2012; Fontana *et al.*, 2015). However, further research is needed to confirm the association of *DRD2 Taq*IA polymorphism with LDF intake and its potential mechanisms.

In summary, our results showed that *Taq*IA polymorphism may have an influence on the children’s eating behavior, due to the presence of the *A1* allele associated with lower D_2_ receptor density that may lead children to compensate the hypodopaminergic functioning with palatable foods. To our knowledge, this is the first association study of the *DRD2 Taq*IA and *-141C Ins/Del* polymorphism with food intake and anthropometric parameters in children at the first stages of development. Notwithstanding, it is necessary to replicate this findings in other populations and identify the mechanisms by which the dopaminergic system may influence food intake. Nevertheless, the investigation of other polymorphisms in this and other genes of the dopaminergic system and their relation to food intake and anthropometric parameters may be interesting.

## References

[B1] Arinami T, Gao M, Hamaguchi H, Toru M (1997). A functional polymorphism in the promoter region of the dopamine D2 receptor gene is associated with schizophrenia. Hum Mol Genet.

[B2] Ariza M, Garolera M, Jurado M, Garcia-Garcia I, Hernan I, Sanchez-Garre C, Vernet-Vernet M, Sender-Palacios MJ, Marques-Iturria I , Pueyo R (2012). Dopamine genes (DRD2/ANKK1-TaqA1 and DRD4-7R) and executive function: their interaction with obesity. PLoS One.

[B3] Barnard ND, Noble EP, Ritchie T, Cohen J, Jenkins DJ, Turner-McGrievy G, Gloede L, Green AA, Ferdowsian H (2009). D2 dopamine receptor Taq1A polymorphism, body weight, and dietary intake in type 2 diabetes. Nutrition.

[B4] Barrett JC, Fry B, Maller J, Daly MJ (2005). Haploview: Analysis and visualization of LD and haplotype maps. Bioinformatics.

[B5] Berenson GS (2012). Health consequences of obesity. Pediatr Blood Cancer.

[B6] Berridge KC (1996). Food reward: Brain substrates of wanting and liking. Neurosci Biobehav Rev.

[B7] Berridge KC, Ho CY, Richard JM, DiFeliceantonio DM (2010). The tempted brain eats: Pleasure and desire circuits in obesity and eating disorders. Brain Res.

[B8] Besnard P (2016). Lipids and obesity: Also a matter of taste?. Rev Endocr Metab Disord.

[B9] Blum K, Noble EP, Sheridan PJ, Montgomery A, Ritchie T, Jagadeeswaran P, Nogami H, Briggs AH, Cohn JB (1990). Allelic association of human dopamine D2 receptor gene in alcoholism. JAMA.

[B10] Briefel RR, Crepinsek MK, Cabili C, Wilson A, Gleason PM (2009). School food environments and practices affect dietary behaviors of US public school children. J Am Diet Assoc.

[B11] Cameron JD, Riou ME, Tesson F, Goldfield GS, Rabasa-Lhoret R, Brochu M, Doucet E (2013). The TaqIA RFLP is associated with attenuated intervention-induced body weight loss and increased carbohydrate intake in post-menopausal obese women. Appetite.

[B12] Cansell C, Luquet S (2016). Triglyceride sensing in the reward circuitry: A new insight in feeding behaviour regulation. Biochimie.

[B13] Carpenter CL, Wong AM, Li Z, Noble EP, Heber D (2013). Association of dopamine D2 receptor and leptin receptor genes with clinically severe obesity. Obesity.

[B14] Cordeiro Q, Siqueira-Roberto J, Zung S, Vallada H (2009). Association between the DRD2-141C Insertion/Deletion polymorphism and schizophrenia. Arq Neuropsiquiatr.

[B15] de Onis M, Blossner M, Borghi E (2010). Global prevalence and trends of overweight and obesity among preschool children. Am J Clin Nutr.

[B16] Du Y, Wan YJ (2009). The interaction of reward genes with environmental factors in contribution to alcoholism in mexican americans. Alcohol Clin Exp Res.

[B17] Dubertret C, Gouya L, Hanoun N, Deybach JC, Ades J, Hamon M, Gorwood P (2004). The 3’ region of the DRD2 gene is involved in genetic susceptibility to schizophrenia. Schizophr Res.

[B18] Duran-Gonzalez J, Ortiz I, Gonzales E, Ruiz N, Ortiz M, Gonzalez A, Sanchez EK, Curet E, Fisher-Hoch S, Rentfro A (2011). Association study of candidate gene polymorphisms and obesity in a young Mexican-American population from South Texas. Arch Med Res.

[B19] Eisenstein SA, Bogdan R, Love-Gregory L, Corral-Frias NS, Koller JM, Black KJ, Moerlein SM, Perlmutter JS, Barch DMc, Hershey T (2016). Prediction of striatal D2 receptor binding by DRD2/ANKK1 TaqIA allele status. Synapse.

[B20] Fontana C, Vitolo MR, Campagnolo PD, Mattevi VS, Genro JP, Almeida S (2015). DRD4 and SLC6A3 gene polymorphisms are associated with food intake and nutritional status in children in early stages of development. J Nutr Biochem.

[B21] Galvão AC, Krüger RC, Campagnolo PDB, Mattevi VS, Vitolo MR, Almeida S (2012). Association of MAOA and COMT gene polymorphisms with palatable food intake in children. J Nutr Biochem.

[B22] Garrido E, Palomo T, Ponce G, Garcia-Consuegra I, Jimenez-Arriero MA, Hoenicka J (2011). The ANKK1 protein associated with addictions has nuclear and cytoplasmic localization and shows a differential response of Ala239Thr to apomorphine. Neurotox Res.

[B23] Gluskin BS, Mickey BJ (2016). Genetic variation and dopamine D2 receptor availability: A systematic review and meta-analysis of human in vivo molecular imaging studies. Transl Psychiatry.

[B24] Grandy DK, Zhang Y, Civelli O (1993). PCR detection of the TaqA RFLP at the DRD2 locus. Hum Mol Genet.

[B25] Gupta N, Goel K, Shah P, Misra A (2012). Childhood obesity in developing countries: epidemiology, determinants, and prevention. Endocr Rev.

[B26] Hardman CA, Rogers PJ, Timpson NJ, Munafo MR (2014). Lack of association between DRD2 and OPRM1 genotypes and adiposity. Int J Obes.

[B27] Himei A, Koh J, Sakai J, Inada Y, Akabame K, Yoneda H (2002). The influence on the schizophrenic symptoms by the DRD2Ser/Cys311 and -141C Ins/Del polymorphisms. Psychiatry Clin Neurosci.

[B28] Hoenicka J, Ponce G, Jimenez-Arriero MA, Ampuero I, Rodriguez-Jimenez R, Rubio G, Aragues M, Ramos JA, Palomo T (2007). Association in alcoholic patients between psychopathic traits and the additive effect of allelic forms of the CNR1 and FAAH endocannabinoid genes, and the 3’ region of the DRD2 gene. Neurotox Res.

[B29] Ishiguro H, Arinami T, Saito T, Akazawa S, Enomoto M, Mitushio H, Fujishiro H, Tada K, Akimoto Y, Mifune H (1998). Association study between the -141C Ins/Del and TaqI A polymorphisms of the dopamine D2 receptor gene and alcoholism. Alcohol Clin Exp Res.

[B30] Johann M, Putzhammer A, Eichhammer P, Wodarz N (2005). Association of the -141C Del variant of the dopamine D2 receptor (DRD2) with positive family history and suicidality in German alcoholics. Am J Med Genet B Neuropsychiatr Genet.

[B31] Jonsson EG, Nothen MM, Neidt H, Forslund K, Rylander G, Mattila-Evenden M, Asberg M, Propping P, Sedvall GC (1999a). Association between a promoter polymorphism in the dopamine D2 receptor gene and schizophrenia. Schizophr Res.

[B32] Jonsson EG, Nothen MM, Grunhage F, Farde L, Nakashima Y, Propping P, Sedvall GC (1999b). Polymorphisms in the dopamine D2 receptor gene and their relationships to striatal dopamine receptor density of healthy volunteers. Mol Psychiatry.

[B33] Kishida I, Kawanishi C, Furuno T, Kato D, Ishigami T, Kosaka K (2004). Association in Japanese patients between neuroleptic malignant syndrome and functional polymorphisms of the dopamine D(2) receptor gene. Mol Psychiatry.

[B34] Konishi T, Calvillo M, Leng AS, Lin KM, Wan YJ (2004a). Polymorphisms of the dopamine D2 receptor, serotonin transporter, and GABA(A) receptor beta(3) subunit genes and alcoholism in Mexican-Americans. Alcohol.

[B35] Konishi T, Luo HR, Calvillo M, Mayo MS, Lin KM, Wan YJ (2004b). ADH1B*1, ADH1C*2, DRD2 (-141C Ins), and 5-HTTLPR are associated with alcoholism in Mexican American men living in Los Angeles. Alcohol Clin Exp Res.

[B36] Lafuente A, Bernardo M, Mas S, Crescenti A, Aparici M, Gasso P, Goti J, Sanchez V, Catalan R, Carne X (2008a). -141C Ins/Del polymorphism of the dopamine D2 receptor gene is associated with schizophrenia in a Spanish population. Psychiatr Genet.

[B37] Lafuente A, Bernardo M, Mas S, Crescenti A, Aparici M, Gasso P, Deulofeu R, Mane A, Catalan R, Carne X (2008b). Polymorphism of dopamine D2 receptor (TaqIA, TaqIB, and-141C Ins/Del) and dopamine degradation enzyme (COMT G158A, A-278G) genes and extrapyramidal symptoms in patients with schizophrenia and bipolar disorders. Psychiatry Res.

[B38] Lahiri DK, Nurnberger JI (1991). A rapid non-enzymatic method for the preparation of HMW DNA from blood for RFLP studies. Nucleic Acids Res.

[B39] Lee SH, Lee BH, Lee JS, Chai YG, Choi MR, Han DM, Ji H, Jang GH, Shin HE, Choi IG (2013). The association of DRD2 -141C and ANKK1 TaqIA polymorphisms with alcohol dependence in Korean population classified by the Lesch typology. Alcohol Alcohol.

[B40] Lencer R, Bishop JR, Harris MS, Reilly JL, Patel S, Kittles R, Prasad KM, Nimgaonkar VL, Keshavan MS, Sweeney JA (2014). Association of variants in DRD2 and GRM3 with motor and cognitive function in first-episode psychosis. Eur Arch Psychiatry Clin Neurosci.

[B41] Lencz T, Robinson DG, Xu K, Ekholm J, Sevy S, Gunduz-Bruce H, Woerner MG, Kane JM, Goldman D, Malhotra AK (2006). DRD2 promoter region variation as a predictor of sustained response to antipsychotic medication in first-episode schizophrenia patients. Am J Psychiatry.

[B42] Lencz T, Robinson DG, Napolitano B, Sevy S, Kane JM, Goldman D, Malhotra AK (2010). DRD2 promoter region variation predicts antipsychotic-induced weight gain in first episode schizophrenia. Pharmacogenet Genomics.

[B43] Lerman C, Jepson C, Wileyto EP, Epstein LH, Rukstalis M, Patterson F, Kaufmann V, Restine S, Hawk L, Niaura R (2006). Role of functional genetic variation in the dopamine D2 receptor (DRD2) in response to bupropion and nicotine replacement therapy for tobacco dependence: results of two randomized clinical trials. Neuropsychopharmacology.

[B44] Li H, Wang X, Zhou Y, Ni G, Su Q, Chen Z, Chen Z, Li J, Chen X, Hou X (2015). Association of LEPR and ANKK1 gene polymorphisms with weight gain in epilepsy patients receiving valproic acid. Int J Neuropsychopharmacol.

[B45] Louzada ML, Campagnolo PD, Rauber F, Vitolo MR (2012). Long-term effectiveness of maternal dietary counseling in a low-income population: A randomized field trial. Pediatrics.

[B46] Miranda RC, Vetter SB, Genro JP, Campagnolo PD, Mattevi VS, Vitolo MR, Almeida S (2015). SLC6A14 and 5-HTR2C polymorphisms are associated with food intake and nutritional status in children. Clin Biochem.

[B47] Mota NR, Araujo-Jr EV, Paixao-Cortes VR, Bortolini MC, Bau CH (2012). Linking dopamine neurotransmission and neurogenesis: The evolutionary history of the NTAD (NCAM1-TTC12-ANKK1-DRD2) gene cluster. Genet Mol Biol.

[B48] Muller DJ, Zai CC, Sicard M, Remington E, Souza RP, Tiwari AK, Hwang R, Likhodi O, Shaikh S, Freeman N (2012). Systematic analysis of dopamine receptor genes (DRD1-DRD5) in antipsychotic-induced weight gain. Pharmacogenomics J.

[B49] Must A (1996). Morbidity and mortality associated with elevated body weight in children and adolescents. Am J Clin Nutr.

[B50] Neville MJ, Johnstone EC, Walton RT (2004). Identification and characterization of ANKK1: a novel kinase gene closely linked to DRD2 on chromosome band 11q23.1. Hum Mutat.

[B51] Oggioni C, Lara J, Wells JC, Soroka K, Siervo M (2014). Shifts in population dietary patterns and physical inactivity as determinants of global trends in the prevalence of diabetes: An ecological analysis. Nutr Metab Cardiovasc Dis.

[B52] Ohara K, Nagai M, Tani K, Nakamura Y, Ino A, Ohara K (1998). Functional polymorphism of -141C Ins/Del in the dopamine D2 receptor gene promoter and schizophrenia. Psychiatry Res.

[B53] Pohjalainen T, Rinne JO, Nagren K, Lehikoinen P, Anttila K, Syvalahti RK, Hietala J (1998). The A1 allele of the human D2 dopamine receptor gene predicts low D2 receptor availability in healthy volunteers. Mol Psychiatry.

[B54] Ponce G, Quinones-Lombrana A, Martin-Palanco MG, Rubio-Solsona E, Jimenez-Arriero MA, Palomo T, Hoenicka J (2016). The addiction-related gene Ankk1 is oppositely regulated by D1R- and D2R-like dopamine receptors. Neurotox Res.

[B55] Popkin BM (2006). Global nutrition dynamics: the world is shifting rapidly toward a diet linked with noncommunicable diseases. Am J Clin Nutr.

[B56] Prasad P, Ambekar A, Vaswani M (2010). Dopamine D2 receptor polymorphisms and susceptibility to alcohol dependence in Indian males: A preliminary study. BMC Med Genet.

[B57] Ritchie T, Noble EP (2003). Association of seven polymorphisms of the D2 dopamine receptor gene with brain receptor-binding characteristics. Neurochem Res.

[B58] Rossner S (1998). Childhood obesity and adulthood consequences. Acta Paediatr.

[B59] Roth CL, Hinney A, Schur EA, Elfers CT, Reinehr T (2013). Association analyses for dopamine receptor gene polymorphisms and weight status in a longitudinal analysis in obese children before and after lifestyle intervention. BMC Pediatr.

[B60] Saiz PA, Garcia-Portilla MP, Arango C, Morales B, Arias B, Corcoran P, Fernandez JM, Alvarez V, Coto E, Bascaran MT (2010). Genetic polymorphisms in the dopamine-2 receptor (DRD2), dopamine-3 receptor (DRD3), and dopamine transporter (SLC6A3) genes in schizophrenia: Data from an association study. Prog Neuropsychopharmacol Biol Psychiatry.

[B61] Salamone JD (1994). The involvement of nucleus accumbens dopamine in appetitive and aversive motivation. Behav Brain Res.

[B62] Stelzel C, Basten U, Montag C, Reuter M, Fiebach CJ (2010). Frontostriatal involvement in task switching depends on genetic differences in d2 receptor density. J Neurosci.

[B63] Stice E, Spoor S, Bohon C, Small DM (2008). Relation between obesity and blunted striatal response to food is moderated by TaqIA A1 allele. Science.

[B64] Suda A, Kawanishi C, Kishida I, Sato R, Yamada T, Nakagawa M, Hasegawa H, Kato D, Furuno T, Hirayasu Y (2009). Dopamine D2 receptor gene polymorphisms are associated with suicide attempt in the Japanese population. Neuropsychobiology.

[B65] Terry P, Gilbert DB, Cooper SJ (1995). Dopamine receptor subtype agonists and feeding behavior. Obes Res 3 (Suppl.

[B66] Ujike H, Katsu T, Okahisa Y, Takaki M, Kodama M, Inada T, Uchimura N, Yamada M, Iwata N, Sora I (2009). Genetic variants of D2 but not D3 or D4 dopamine receptor gene are associated with rapid onset and poor prognosis of methamphetamine psychosis. Prog Neuropsychopharmacol Biol Psychiatry.

[B67] van Strien T, Snoek HM, van der Zwaluw CS, Engels RC (2010). Parental control and the dopamine D2 receptor gene (DRD2) interaction on emotional eating in adolescence. Appetite.

[B68] Vitolo MR, Rauber F, Campagnolo PD, Feldens CA, Hoffman DJ (2010). Maternal dietary counseling in the first year of life is associated with a higher healthy eating index in childhood. J Nutr.

[B69] Volkow ND, Wang GJ, Fowler JS, Logan J, Jayne M, Franceschi D, Wong C, Gatley SJ, Gifford AN, Ding YS (2002). “Nonhedonic” food motivation in humans involves dopamine in the dorsal striatum and methylphenidate amplifies this effect. Synapse.

[B70] Wang GJ, Volkow ND, Logan J, Pappas NR, Wong CT, Zhu W, Netusil N, Fowler JS (2001). Brain dopamine and obesity. Lancet.

[B71] Wang Y, Liu L, Xin L, Fan D, Ding N, Hu Y, Cai G, Wang L, Xia Q, Li X (2016). The -141C Ins/Del and Taq1A polymorphism in the dopamine D2 receptor gene may confer susceptibility to schizophrenia in Asian populations. J Clin Neurosci.

[B72] Winkler JK, Woehning A, Schultz JH, Brune M, Beaton N, Challa TD, Minkova S, Roeder E, Nawroth PP, Friederich HC (2012). TaqIA polymorphism in dopamine D2 receptor gene complicates weight maintenance in younger obese patients. Nutrition.

[B73] Wu S, Xing Q, Gao R, Li X, Gu N, Feng G, He L (2005). Response to chlorpromazine treatment may be associated with polymorphisms of the DRD2 gene in Chinese schizophrenic patients. Neurosci Lett.

[B74] Xiao L, Shen T, Peng DH, Shu C, Jiang KD, Wang GH (2013). Functional -141C Ins/Del polymorphism in the dopamine D2 receptor gene promoter and schizophrenia in a Chinese Han population. J Int Med Res.

[B75] Zhang JP, Lencz T, Malhotra AK (2010). Dopamine D2 receptor genetic variation and clinical response to antipsychotic drug treatment: A meta-analysis. Am J Psychiatry.

[B76] Zhao X, Huang Y, Chen K, Li D, Han C, Kan Q (2016). -141C insertion/deletion polymorphism of the dopamine D2 receptor gene is associated with schizophrenia in Chinese Han population: Evidence from an ethnic group-specific meta-analysis. Asia Pac Psychiatry.

